# Development of Co-Amorphous Systems for Inhalation Therapy—Part 2: In Silico Guided Co-Amorphous Rifampicin–Moxifloxacin and –Ethambutol Formulations

**DOI:** 10.3390/pharmaceutics17101339

**Published:** 2025-10-16

**Authors:** Eleonore Fröhlich, Noon Sharafeldin, Valerie Reinisch, Nila Mohsenzada, Stefan Mitsche, Hartmuth Schröttner, Sarah Zellnitz-Neugebauer

**Affiliations:** 1 Research Center Pharmaceutical Engineering, Inffeldgasse 13, 8010 Graz, Austria; eleonore.froehlich@medunigraz.at (E.F.);; 2 Center for Medical Research, Medical University of Graz, Stiftingtalstr. 24, 8010 Graz, Austria; 3 Institute of Electron Microscopy and Nanoanalysis (FELMI), Graz University of Technology, Steyrergasse 17, 8010 Graz, Austria; stefan.mitsche@felmi-zfe.at (S.M.); hartmuth.schroettner@felmi-zfe.at (H.S.); 4 Graz Centre for Electron Microscopy (ZFE), Steyrergasse 17, 8010 Graz, Austria

**Keywords:** co-amorphous, inhalation, tuberculosis, ethambutol, rifampicin, moxifloxacin, PBPK modeling

## Abstract

**Background/Objectives:** Tuberculosis (TB) remains a global health challenge due to long treatment durations, poor adherence, and growing drug resistance. Inhalable co-amorphous systems (COAMS) offer a promising strategy for targeted pulmonary delivery of fixed-dose combinations, improving efficacy and reducing systemic side effects. **Methods**: Our in-house-developed machine learning (ML) tool identified two promising API-API combinations for TB therapy, rifampicin (RIF)–moxifloxacin (MOX) and RIF–ethambutol (ETH). Physiologically based pharmacokinetic (PBPK) modeling was used to estimate therapeutic lung doses of RIF, ETH, and MOX following oral administration. Predicted lung doses were translated into molar ratios, and COAMS of RIF-ETH and RIF-MOX at both model-predicted (1:1) and PBPK-informed ratios were prepared by spray drying and co-milling, followed by comprehensive physicochemical and aerodynamic characterization. **Results**: RIF-MOX COAMS could be prepared in all molar ratios tested, whereas RIF-ETH failed to result in COAMS for therapeutically relevant molar ratios. Spray drying and ball milling successfully produced stable RIF-MOX formulations, with spray drying showing superior behavior in terms of morphology (narrow particle size distribution; lower Sauter mean diameter), aerosolization performance (fine particle fraction above 74% for RIF and MOX), and dissolution. **Conclusions**: This study demonstrated that PBPK modeling and ML are useful tools to develop COAMS for pulmonary delivery of active pharmaceutical ingredients (APIs) routinely applied through the oral route. It was also observed that COAMS may be less effective when the therapeutic lung dose ratio significantly deviates from the predicted 1:1 molar ratio. This suggests the need for alternative delivery strategies in such cases.

## 1. Introduction

Tuberculosis (TB) is a major global health burden caused by *Mycobacterium tuberculosis* and is traditionally managed using oral and parenteral administration of antibiotics. The treatment is complex and requires a multi-drug regimen therapy of different drugs over a long periods of time [1]. The World Health Organization (WHO)’s supervisory treatment strategy recommends multi-drug treatment in different phases. In the initial intensive phase, a combination of first-line drugs (isoniazid, rifampicin, pyrazinamide, ethambutol, and streptomycin) for at least 2 months is required. In the continuation phase, a combination of two or three second-line drugs (ethionamide, prothionamide, kanamycin, amikacin, capreomycin, terizidone, cycloserine, viomycin, and para-amino salicylic acid) is used for at least 4 months to sterilize lesions [2]. The treatment of TB remains complex and is often accompanied by antibiotic resistance. In response to these challenges, the early 2000s marked a renewed focus on developing novel therapies and treatment regimens for TB. New antibiotics, including bedaquiline and moxifloxacin [3,4], have been introduced, and clinical guidelines have been updated to differentiate between drug-susceptible and drug-resistant TB, allowing for more targeted treatment strategies. [5,6], tailoring treatment accordingly. Yet, even with these advances, therapeutic outcomes are often suboptimal, and innovation in delivery methods is urgently needed.

While current systemic therapies remain effective, they present several limitations. Oral regimens typically require extended treatment durations, which can lead to poor adherence, gastrointestinal adverse effects, and systemic toxicity. Parenteral administration introduces additional challenges, including patient discomfort, logistical complexity, and increased healthcare burden. Moreover, both routes often fail to achieve optimal drug concentrations in pulmonary tissues—the primary site of *Mycobacterium tuberculosis* infection. Inhalation therapy offers a promising alternative by enabling direct drug delivery to the lungs, with the potential to enhance local drug concentrations, minimize systemic exposure, and improve overall treatment outcomes [7,8,9].

Considerable research over the past two decades has focused on inhalation-based therapies for TB. Various approaches have been explored, including the development of dry powder inhalers (DPIs) via spray drying (SD) [10], liposomal formulations [11,12], and nanoparticle-based carriers [13,14,15].

Rifampicin (RIF) has demonstrated favorable aerosolization properties when SD or freeze-dried, with reported fine particle fraction (FPF) values of around 70% [16]. However, to date, only one notable study has evaluated inhaled RIF in humans. Srichana et al. assessed the safety and tolerability of a low-dose (2 mg) DPI formulation in 39 healthy volunteers, confirming the safety of RIF alongside other first-line anti-TB drugs such as isoniazid, pyrazinamide, and levofloxacin [17]. Despite these promising findings, key challenges remain—most notably, the accurate estimation of therapeutic inhaled doses required to achieve plasma levels comparable to high-dose oral administration. Inhalation offers the potential for significantly reduced doses while mitigating systemic side effects associated with oral antibiotics.

While ingesting several tablets is usually not a major problem, patients do not easily accept inhaling several times, potentially with different inhalers. The introduction of fixed-dose combinations to the market has improved the treatment of obstructive lung diseases, e.g., for obstructive pulmonary disease, but the generation is not trivial [16]. Among the techniques to generate formulations containing several active pharmaceutical ingredients (APIs), the generation of fixed-dose combinations by creating co-amorphous systems (COAMS) is less commonly used. Typically, COAMS are generated via mechanical milling, solvent evaporation (including freeze-drying and SD), and melt quenching or quench cooling [17], and the advantages associated with COAMS comprising two APIs for inhalation therapy are the minimized variability in drug doses when administering combination products and more lean and economical manufacturing. However, the formulation must be designed so that it delivers the correct amount of each API to the lungs. A common approach has been to administer an inhaled dose equivalent to one-tenth of the oral dose [10]; however, this method does not account for drug-specific pharmacokinetics and dynamics. Both experimental approaches and theoretical calculations suggest that stable COAMS can be produced by similar molecular API ratios because various molecular interactions are possible [18].

Recent studies, such as the development of age-appropriate fixed-dose combinations of isoniazid and RIF using physiologically based pharmacokinetic (PBPK) modeling in children, highlight how model-informed approaches can improve dosing strategies across different patient groups [19]. Building on this concept, we employed PBPK modeling using the GastroPlus™ (GP) stimulation software to estimate lung doses for three classical anti-TB drugs, RIF, ethambutol (ETH), and moxifloxacin (MOX) upon oral exposure. In a second step, we calculated which ratios between the APIs result in the same relative tissue concentrations when APIs are administered by a pulmonary formulation with FPF of 40%. Using an in-house-developed model, drug combinations for COAMS were identified. This model uses machine learning (ML) based on 35 descriptors and predicted correct API-API pairs with 79% accuracy. More details can be found in Fink et al. [18]. Building on that foundation, we identified promising combinations for TB treatment such as RIF-ETH and RIF-MOX, the latter being relevant for multidrug-resistant (MDR) TB [4]. While our model predicted a 1:1 molar ratio for the APIs, this may not reflect the therapeutically needed ratio in lung tissue.

Therefore, the present study focuses on the preparation of COAMS of RIF–ETH and RIF–MOX at therapeutically relevant molar ratios. In a first step, we estimate lung doses using PBPK modeling. In the next step, COAMS of RIF-ETH and RIF-MOX in the ratios predicted by our model or by PBPK modeling were produced by co-milling (CM) and SD. The powders were characterized according to their solid state, particle morphology, aerodynamic performance, and dissolution behavior.

## 2. Materials and Methods

### 2.1. Materials

Rifampicin and ethambutol dihydrochloride were purchased from TCI Deutschland GmbH (Eschborn, Germany) and moxifloxacin hydrochloride from Shenzhen Nexconn Pharmatechs Ltd. (Shenzhen, China).

Solvents used for analytics (formic acid; methanol) and SD (absolute ethanol) were purchased from Lactan Chemikalien and Laborge-raete Vertriebsgesellschaft m.b.H & Co. KG (Graz, Austria).

For the preparation of simulated lung fluid (SLF), sodium chloride (Merck KGaA, Darmstadt, Germany), potassium chloride (Carl Roth, Karlsruhe, Germany), di-sodium hydrogen phosphate (Carl Roth), potassium di-hydrogen phosphate (Carl Roth), and 1,2-Dipalmitoyl-sn-glycero-3-phosphocholine (DPPC) (TCI Deutschland GmbH, Germany) were used. For aerosolization tests, hard-gelatin Coni-Snap^®^ size 3 capsules provided by Capsugel (Cologne, Germany) were used, and a capsule-based inhaler, the Cyclohaler^®^ (PB Pharma GmbH, Meerbusch, Germany), was bought at a local pharmacy.

### 2.2. Modeling to Obtain Physiological Lung Doses

We developed a ML model to predict COAMS between two APIs used in inhalation therapy. The model showed 79% accuracy, and further validation yielded an experimental accuracy of 72% [20]. COAMS predictions were based on a 1:1 molar ratio, selected for its prevalence in the literature and stoichiometric preference, despite limited therapeutic relevance. To translate these findings into clinically meaningful ratios, two approaches are proposed: (1) applying the general rule that inhalation doses are ~10× lower than oral doses [10], and (2) using GP modeling to estimate lung doses from oral administration, enabling more accurate therapeutic molar ratios.

In silico modeling and simulation were performed using GastroPlus™ version 9.8 (SimulationPlus, Inc., Durham, NC, USA) to evaluate the pharmacokinetic behavior of the selected APIs. For each API, a PBPK model was developed to simulate drug concentration profiles within the human body and to estimate pharmacokinetic (PK) parameters. These simulated parameters were then compared to values reported in clinical studies or available in the literature to assess model accuracy and predictive capability.

The molecular structures of the APIs were first imported into GP. Default physiological and physicochemical parameters were applied where applicable, unless the literature values were available. Tissue-to-plasma partition coefficients (Kp values) were estimated using the Rodgers and Rowland method selected in GP; these were extracted and then scaled to the volume of distribution at steady state (Vd_ss_) [21] (Table 1). Additional key input parameters, including the effective permeability (P_eff_) and intravenous clearance (CL_iv_), were taken from sources in the literature and are summarized in Table 2.

All simulations were performed using the study’s mean anthropometric parameters when available. In the case of RIF, a standardized virtual individual from GP was used. When study-reported anthropometric data were available, mean values were used to define the virtual individual. In cases where the study population included a mix of sexes or races, a representative profile was selected for simulation purposes. Specifically, a male Caucasian virtual individual was used consistently across simulations to maintain uniformity, acknowledging that some study populations (e.g., for ethambutol) included both male and female participants, as well as individuals of different racial backgrounds. This approach was necessary due to the use of a single virtual subject per compound. Mean anthropometric parameters of the study population are summarized in Table 3.

Simulated plasma concentration–time profiles were validated by comparison with observed data from published sources. If the predicted pharmacokinetic parameters—such as peak concentration (*Cmax*) and area under the curve (*AUC*)—fell within ±20% of the literature values, the simulation was considered sufficiently accurate to proceed to lung tissue concentration modeling.

To quantify pulmonary exposure, the area under the concentration–time curve in lung tissue (AUC_lung_) was calculated based on simulated concentration profiles from the PBPK model. The resulting values, expressed in µg·h/mL, were then multiplied by the lung tissue volume (V_lung_) to estimate the total exposure in terms of drug mass over time (µg·h). This value represents the cumulative presence of the API in the pulmonary compartment throughout the simulated period. By dividing this value by the administered dose (µg), the fraction of the dose retained in the lung over time was estimated. This serves as a surrogate metric for evaluating pulmonary targeting efficiency and can support the rational design of inhaled dosage forms.

$$\mathrm{Lung}\;\mathrm{Exposure}\;\left(\%\right)=\frac{{\mathrm{AUC}}_{\mathrm{lung}}\cdot V_{\mathrm{lung}}}{\mathrm{Administered}\;\mathrm{dose}}\cdot 100$$

### 2.3. Translating Calculated/Predicted Estimated Lung Exposure to Dose

The inhalation doses of RIF, ETH, and MOX were calculated based on the common oral therapeutic doses of each API (600 mg, 1200 mg, and 400 mg, respectively [6]), the estimated lung exposure from GP modeling, and the overall reduced percentage of inhaled dose that reaches the lung during inhalation (FPF). Based on previous data [20], FPF was fixed to 40%. Additionally, an inhalation dose based on a literature-estimated lung exposure ~10× lower than the oral dose [10] was tested.

$$\frac{\mathrm{Oral}\;\mathrm{Dose}\cdot \mathrm{Lung}\;\mathrm{exposure}\left(\%\right)}{\mathrm{FPF}}=\mathrm{Inhalation}\;\mathrm{Dose}$$

### 2.4. Preparation of COAMS

To prepare COAMS of RIF-ETH and RIF-MOX, CM and SD were chosen. For CM, the two APIs were placed in the respective molar ratios in a 50 mL stainless steel vessel together with 10 stainless steel grinding balls (diameter, 10 mm). Milling was performed in a planetary ball mill (Pulverisette 6, Fritsch GmbH, Anthering, Austria) in 25 min milling cycles with a 2 min pause in between and repeated 8 times for RIF-ETH and 6 times for RIF-MOX at 650 rpm, with the rotation direction reversing after each milling cycle. Additionally, RIF-MOX (therapeutically relevant molar ratios) combinations were jet-milled in a Hosokawa Spiral Airjetmill 50 AS (HOSOKAWA ALPINE Aktiengesellschaft, Augsburg, Germany), with an inlet air pressure of 6 bar and a milling air pressure of 3 bar. Although all samples were CM, in the following sections, the ball-milled samples alone are called BM, and the ball-milled plus jet-milled samples are called CM (for co-milled).

SD was performed using a BUCHI Mini Spray Dryer B-290 (Buchi Labortechnik AG, Flawil, Switzerland) in a closed-loop configuration using nitrogen gas. To prepare the feed solution, RIF-ETH combinations were dissolved in an ethanol–water mixture (80:20 *v*/*v*) with a solid concentration of 4% (*w*/*v*) and RIF-MOX combinations in an ethanol–water mixture (60:40 *v*/*v*) with a solid concentration of 2% (*w*/*v*). The following SD conditions were used: inlet temperature—90 °C, airflow rate—1052 L/h, aspiration—100%, pump speed—10%, and nozzle cap—1.4 mm. Particles were collected in a collecting container using a high-performance cyclone.

The different API-API combinations, the methods used to prepare them, and the respective molar ratios at which they were tested are summarized in Table 4.

As a reference, physical mixtures (PMs) of micronized RIF and MOX in therapeutically relevant molar ratios, a total of 1 g each, were mixed in a tumble blender (T2F Turbula^®^, Willy A. Bachofen AG Maschinenfabrik, Muttenz, Switzerland) at 32 rpm for 30 min and then sieved through a 400 µm sieve and mixed again for 10 min. Micronization of RIF and MOX was performed with a jet mill (Hosokawa Spiral Airjetmill 50 AS). The powder was slowly manually fed, and an inlet pressure of 6 bar and a milling pressure of 3 bar were set.

All samples were stored dry in a desiccator over silica gel at room temperature (25 ± 5 °C) after preparation and only removed for different analyses for the minimum time required.

### 2.5. Particle Characterization

#### 2.5.1. Solid State—XRD Measurements

The co-processed RIF-ETH and RIF-MOX samples, along with the PM of RIF-MOX and the individual pure compounds RIF, ETH, and MOX, were analyzed by X-ray diffraction (XRD) shortly after preparation (within approximately one hour of milling). The analysis was conducted using a Siemens D5005 diffractometer (Siemens AG, Munich, Germany) configured in Bragg–Brentano geometry with a copper anode (λ = 1.54186 Å), operating at 40 kV and 40 mA. Data were collected over a 2θ range of 4° to 40°, using a step size of 0.04° and a dwell time of 2 s per step. To count the X-rays, a scintillation detector was used. In the Results and Discussion section, co-processed samples that no longer show characteristic crystalline Bragg peaks in XRD patterns are referred to as amorphous.

#### 2.5.2. Particle Size Measurements—HELOS

Laser diffraction particle sizing was carried out using a Sympatec HELOS KR instrument with a Sympatec KUVETTE dispersing unit (Sympatec GmbH, Clausthal-Zellerfeld, Germany) fitted with R2 and R5 measuring lenses (0.45–875 μm) and a magnetically stirred cell. A concentrated suspension of samples in silicon oil was created by adding one spatula to around 2 mL of silicon oil (SILIKONÖL B2 (2 cst, QUAX GmbH, Obernburg, Germany) and then sonicated for approximately 30 s in an ultrasonic bath. A background reading was taken from the pure silicon oil before each measurement, and then, the suspension was added dropwise to the cuvette (filled with 50 mL of silicon oil) to reach an optical concentration of around 15%. The sample cuvette was stirred at 800 RPM; measurement time was 10 s. For each sample, 3 measurements were conducted (*n* = 3) to obtain particle size measurements (X_10_, X_50_, and X_90_, corresponding to the particle sizes below which 10%, 50%, and 90% of the particles are smaller than by number, and the Sauter mean diameter (SMD), as well as the % of particles below 5 µm). Data evaluation was performed using the software WinDox 5.6.0.0 (Sympatec, Clausthal-Zellerfeld, Germany), and particle sizes were calculated using the Fraunhofer theory.

#### 2.5.3. Particle Morphology—SEM Images

Scanning electron microscopy (SEM) was employed to examine the surface morphology of the pure active pharmaceutical ingredients (RIF, ETH, and MOX), the co-processed RIF-ETH and RIF-MOX samples, and the PM of RIF-MOX. The analysis was performed using a Zeiss Ultra 55 microscope (Zeiss, Oberkochen, Germany). Prior to imaging, all samples were gold–palladium-sputtered, and the microscope was operated at an accelerating voltage of 5 kV.

#### 2.5.4. Stability Study

To assess the stability of the COAMS, the samples were kept in desiccators over silica gel at ambient temperature (25 ± 3 °C), and after 3 months, XRD measurements were conducted to obtain information on the amorphous and crystalline state.

### 2.6. Particle Performance

#### 2.6.1. API Distribution/API Content

The uniformity of API content in the PM, SD, and CM RIF-MOX COAMS formulations was assessed by analyzing 10 individual samples of approximately 5 mg each. Drug content was determined using high-performance liquid chromatography (HPLC; Section 2.7). Samples were dissolved in 25 mL of the appropriate HPLC mobile phase, and the average drug content is reported as the percentage mean across the 10 samples, while mixing homogeneity is indicated by the relative standard deviation of the mean drug content among the samples.

#### 2.6.2. Aerosolization Performance

The aerodynamic performance of the formulations was evaluated using a Fast-Screening Impactor (FSI) (Copley Scientific, Nottingham, UK) in combination with the Cyclohaler^®^, a capsule-based, low-resistance DPI. Capsules were manually filled with 25 ± 2 mg of drug-only formulation. The testing procedure followed the European Pharmacopoeia guidelines for inhalation preparations: aerodynamic assessment of fine particles (Ph. Eur., 7.0). The FSI separates aerosol particles into two size-based fractions: those with an aerodynamic diameter greater than 5 µm are collected in the induction port and preseparator, while those smaller than 5 µm are captured in the fine fraction collector (FFC) on a glass fiber filter. An airflow rate of 60 L/min was applied during testing to achieve a pressure drop of 4 kPa, using the corresponding CFC insert suitable for this flow rate. The airflow was maintained for 4 s to ensure a total air volume of 4 L passed through the inhaler via the mouthpiece. Drug deposition on each component of the impactor (mouthpiece, induction port, preseparator, FFC, and the inhaler with the capsule) was quantified using a validated analytical method (see Section 2.8).

Each formulation was tested in triplicate. To assess performance, two key parameters were used: the emitted fraction (EF) and FPF. EF (%) represents the percentage of API recovered from the impactor (induction port, CFC, and FFC) relative to the recovered dose. FPF (%) indicates the proportion of API with an aerodynamic diameter less than 5 µm, calculated as the ratio of fine particle mass (FPM, i.e., the mass collected in the FFC) to the emitted dose (ED).

#### 2.6.3. Dissolution Studies

Dissolution studies were conducted over a 180 min period using simulated lung fluid (SLF) as the dissolution medium. SLF was prepared by dissolving 40 mg of DPPC in 3.076 mL of ethanol; then, the solution was slowly added to 200 mL of preheated phosphate-buffered saline (PBS) at approximately 37 °C under continuous stirring. The resulting slightly turbid mixture was placed in an ultrasonic bath at 37 °C for about 20 min until a clear solution was obtained. The PBS buffer was prepared by adding 1600 mg of sodium chloride, 288 mg of di-sodium hydrogen phosphate, 40 mg of potassium chloride, and 48 mg of potassium di-hydrogen phosphate to a 200 mL volumetric flask and filling it up with Milli-Q water.

For the dissolution tests, FSI was performed as mentioned in Section 2.6.2 and the filters from the FFC, which capture particles < 5 µm, were placed in a 250 mL crystallizing dish covered with aluminum foil to block light, to which 55 mL of SLF was added. The dish was then placed on an incubator shaker set to 37 °C and 60 RPM. While several methodologies for evaluating the dissolution of inhalable formulations exist in the literature, a universally accepted standard has not yet been established [28]. The method used here is a modified setup designed to better simulate physiological conditions, particularly by limiting the volume of the dissolution medium. Sampling was performed immediately (0 min) and at 2, 5, 10, 20, 40, 60, 120, and 180. At each time point, 1000 µL of the dissolution medium was withdrawn and immediately replaced with equally fresh SLF to maintain a constant volume. All experiments were carried out in triplicate. Collected samples were filtered through a 0.22 nylon filter into HPLC vials and analyzed for drug content using HPLC (Section 2.7).

For dissolution tests, 1 capsule containing 25 mg of formulation was used. This corresponds to a theoretical dose of around 15 mg of RIF and 10 mg of MOX for RIF-MOX 1:1.25 and 2 mg of RIF and 23 mg of MOX for RIF-MOX 1:23.8.

### 2.7. HPLC Analysis

Quantification of RIF and MOX was performed using reverse-phase ultra-performance liquid chromatography (RP-UPLC) on an Acquity UPLC H-Class^®^ system (Waters Corp., Milford, MA, USA) equipped with a photodiode array (PDA) detector and operated with the Empower 3 chromatography software (Version 7.50.00.00). The analytical method details are found in Table 5.

All samples and calibration standards were prepared in methanol as a diluent and filtered through 0.22 µm nylon filters prior to analysis. The method was verified for linearity in a range of 5–200 µg/mL for both analytes.

### 2.8. Statistical Evaluation

Differences between the formulations (RIF-MOX PM, RIF-MOX CM, and RIF-MOX SD) regarding key parameters, like X_10_, X_50_, X_90_, FPF, and EF, were statistically analyzed using a one-way ANOVA test. Once a significant difference was found using ANOVA, a two-tailed t-test of means (two-sample assuming unequal variances) was performed between pairs of the three formulations to determine which formulations differed significantly. The Microsoft Excel software was used for the ANOVA and *t*-test, and the significance level was set at 0.05 (*p* = 0.05) for both tests.

## 3. Results and Discussion

### 3.1. Physiological Lung Doses

When inputting the values from Table 1 and Table 2 into the GP model, the resulting simulated PK-parameters are presented in Table 6.

**Figure 1:**
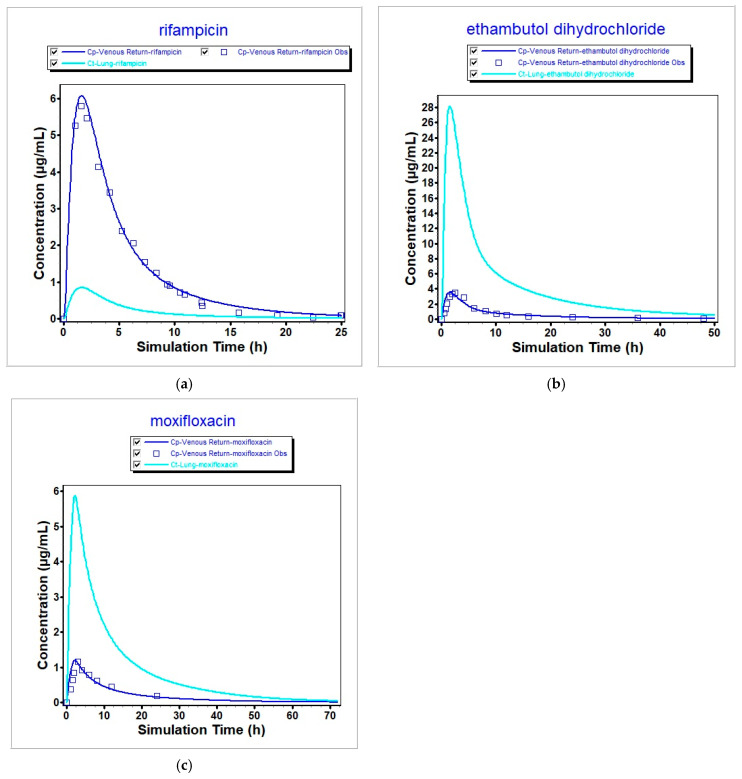
Concentrations in plasma (dark blue line) and lung tissue (bright blue line) of (**a**) RIF, (**b**) ETH, and (**c**) MOX, taken from [22], [23], and [24], respectively.

The corresponding concentration–time profiles are shown in Figure 1, where the blue squares represent the mean profiles obtained from the clinical trial data (RIF, ETH, and MOX taken from [22], [23], and [24], respectively), the dark blue line represents the simulated plasma concentration profile, and the bright blue represents the predicted lung tissue concentration. We validate our model with human PK values from the literature (Table 6), where, for all the parameters, the percentage prediction error (%PE) is less than or equal to 10%, and compare lung predictions against human pulmonary matrices (tissue/bronchial mucosa/lesion data), where available. For RIF, patient positron emission tomography (PET) with [^11^C] rifampin, a radioactive marked version of the same molecule, showed a tissue-to-plasma AUC well below 1 (about 0.30 ± 0.07 in cavity walls), so it is plausible that lung tissue exposure can fall under plasma [29]. For MOX, healthy-volunteer bronchoscopy shows a bronchial–mucosa AUC over 0–24 h of roughly 1.8× plasma [30], and surgical microdialysis in TB patients shows median free tissue of about 3.2× free serum [31], consistent with enrichment, as is also visible in our simulations. ETH likewise concentrates in pulmonary compartments [32]. As can be seen in Figure 1, these findings match our qualitative conclusion, even though bulk tissue AUC data are sparse.

The simulated tissue profiles (Figure 1) allow for the estimation of drug exposure in the lung compartment. The final estimated pulmonary drug exposures for the compounds of interest are presented in Table 7.

The estimated lung exposure from Table 7 was transferred to an inhalation dose for each API (Table 8).

The therapeutic molar ratios corresponding to the inhalation dose of RIF-ETH and RIF-MOX based on the GP model can be seen in Table 9 and were calculated based on each API’s molar mass (see Section 2.2. Modeling to obtain physiological lung doses—Table 2). The inhalation dose (the literature) and molar ratio (the literature), corresponding to an estimated lung exposure ~10× lower than the oral dose [10], are also presented in Table 8 and Table 9.

Factors related to translating the knowledge about the relative changes in drug concentrations of orally applied APIs to an effective inhaled dose, e.g., deposition efficiency, pulmonary absorption, and drug clearance, are beyond the scope of this study.

Nevertheless, some considerations are necessary to frame the limitations of estimating lung doses from oral data. Estimating lung doses for inhaled formulations based on oral dosing data is not straightforward and comes with notable limitations. Oral administration leads to systemic distribution, with only a small and variable fraction reaching the lungs. Furthermore, metabolization by the liver may take place. The rule that one tenth of the oral dose is appropriate for pulmonary application depends on the physicochemical characteristics of the API, the formulation, and the disease. For antibiotics, such as ciprofloxacin, 10–100-times-higher lung tissue concentrations for inhaled formulations compared to oral formulations have been determined [33]. For the generation of fixed-dose formulations for inhalation, the fraction of orally administered APIs that reach the lung is particularly important. As an approximation, it was assumed that permeation across the gastrointestinal mucosa (e.g., mucus permeation and epithelial layer) shows no major differences from the permeation of the respiratory epithelium. We are aware that, for the application in TB, lung tissue levels are heterogeneous, especially in patients with poorly vascularized lung lesions [28]. Consequently, it is unknown how the ratios between the APIs are changed in these regions.

The concentration of 2.3 µg/g lung tissue for an oral dose of 600 mg reported by Kiss et al. [34] corresponds to a 0.38 µg/100 mg RIF dose. The concentration of oral MOX in human lung tissue was reported as 16.2 µg/g for a dose of 400 mg or 4 µg/100 mg MOX [35]. Considering the respective molecular weights of the APIs, the molecular ratio of MOX/RIF is 21.5, which is close to the predicted one and may serve as independent validation of the in silico simulations. No experimental data for lung concentrations were available for ETH.

### 3.2. Particle Characterization

#### 3.2.1. Solid State

**Figure 2:**
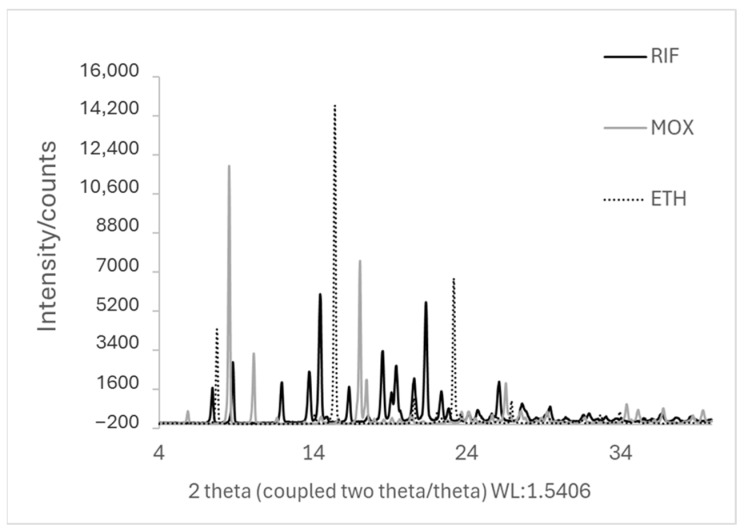
XRD pattern of the starting materials (jet-milled RIF, ETH, and MOX).

The results in Figure 2 show the crystalline nature of the starting materials (RIF, ETH, and MOX). RIF exhibits several characteristic peaks at 7.36°, 8.70°, 13.66°, 14.38°, and 21.26 [36], while ethambutol (ETH) displays three main peaks at 7.60°, 15.30°, and 23.0°, confirming its crystalline structure [37].

**Figure 3:**
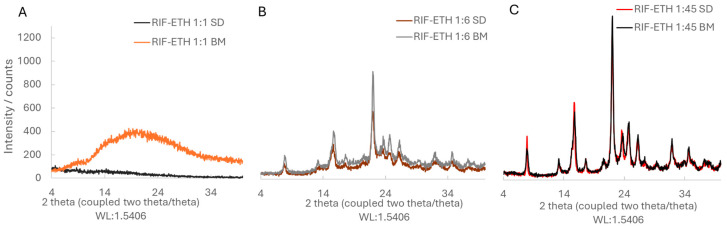
XRD patterns of the different RIF-ETH formulations: (**A**) spray-dried (SD) and ball-milled (BM) in a molar ratio of 1:1; (**B**) SD and BM in a molar ratio of 1:6; (**C**) SD and BM in a molar ratio of 1:45.

**Figure 4:**
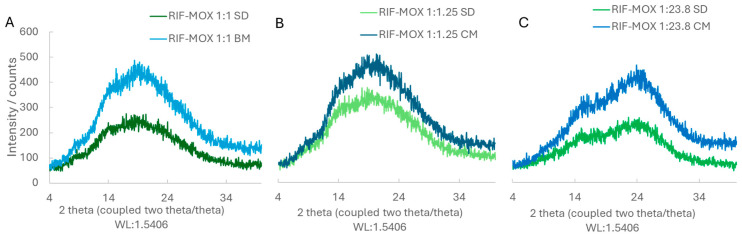
XRD patterns of the different RIF-MOX formulations: (**A**) spray-dried (SD) and ball-milled (BM) in a molar ratio of 1:1; (**B**) SD and BM in a molar ratio of 1:1.25; (**C**) SD and BM in a molar ratio of 1:23.8.

As established in our previous work, for COAMS generation, CM and SD were selected. The XRD results show that in a molar ratio of 1:1, RIF-ETH and RIF-MOX are co-amorphous (Figure 3A and Figure 4A, respectively). This is in accordance with our ML model prediction. RIF-ETH 1:1 had a predictability of 1 and a distance from the training dataset of 361, and RIF-MOX 1:1 has a predictability of 1 and a distance from the training dataset of 16. The formation of COAMS is predicted with a prediction value close to 1 (a value of 1 means 100% certainty to be co-amorphous) and a low distance value. More details on the model and predictability are presented in part 1 of the current paper (Development of Co-Amorphous Systems for Inhalation Therapy—Part 1: From Model Prediction to Clinical Success [20]) and in our previous paper on the predictive ML model [38]. However, when testing the new therapeutically relevant molar ratios, RIF-ETH combinations fail to result in COAMS (Figure 3B,C). For both molar ratios tested, 1:6 and 1:45, crystalline peaks can still be observed in XRD images, irrespective of the preparation method used, SD versus CM. Compared to the crystalline starting materials (Figure 2), in the CM and SD RIF-ETH samples, peak intensity is drastically reduced (see y-axis scale, Figure 2 and Figure 3B,C); however, crystalline peaks, mainly from ETH, can still be detected. An overlay of the peaks with the starting materials is provided in the Supplementary Materials (Figure S1).

By contrast, for both RIF-MOX samples in molar ratios of 1:1.25 and 1:23.8, COAMS were formed (Figure 4B,C), which is evident in the XRD patterns shown in Figure 4. Instead of sharp, characteristic Bragg peaks, as observed for the pure crystalline substances in Figure 3, the diffraction pattern displays only a broad amorphous halo, which indicates the absence of long-range order. This is independent of the co-amorphization approach used and true for CM and SD. One possible explanation is that the distance value of the two formulations was much lower for RIF-MOX. This means that the formation of COAMS is still predicted when the molar ratio deviates significantly from the predicted 1:1 ratio.

To prepare RIF-MOX PMs, jet-milled starting materials (RIF and MOX) were used. XRD images of the PMs of RIF-MOX 1:1.25 and 1:23.8 show characteristic peaks of both APIs (RIF and MOX), confirming that both APIs are still present in their crystalline form. Graphs are shown in Supplementary Materials Figure S2.

The successful formation of RIF-ETH COAMS at a molar ratio of 1:1, but not at other tested therapeutically relevant molar ratios, confirms once again that intermolecular interactions such as hydrogen bonds play a crucial role in stabilizing the amorphous state. A 1:1 ratio often provides the optimal stoichiometry for complementary interaction sites and enables a balanced and saturated hydrogen bonding network. At non-equimolar ratios, excess molecules may not be sufficiently stabilized, increasing the likelihood of recrystallization. In fact, the most commonly used and described molar ratio in the literature is 1:1. However, Liu et al. reported that a molar ratio of 1:1 is probably not always the ideal molar ratio in terms of the physical stability and/or dissolution behavior of COAMS [17]; instead, this depends heavily on the compounds used. However, only a few studies report on the optimization of the molar ratio [39]. In our case, the molar ratio appears to be more decisive for RIF-ETH, but not for RIF-MOX. As already mentioned, this could also be related to the distance value provided by the model.

Since RIF-ETH formulations at therapeutically relevant ratios were not co-amorphous, no further optimization of the SD process or additional jet milling was performed to generate powders with suitable properties for inhalation.

#### 3.2.2. Particle Size Measurements

Particle size analysis of RIF-ETH (Table 10) formulations revealed BM did not yield particles suitable for inhalation with a size below 5 µm. Also, the SPAN values are generally higher for the BM samples, indicating a broader particle size distribution. This is also reflected in the extremely high values for x_90_. One possible explanation is that the particles adhered to the walls of the milling vessel. There, they were pressed by the grinding balls and could not be milled efficiently.

SD yielded particles suitable for inhalation in molar ratios of 1:1 and 1:45, with, respectively, an x_50_ of 2.1 µm, a SPAN of 0.98, and an SMD of 2.17 µm and an x_50_ of 3.77 µm, a SPAN of 1.57, and an SMD of 2.63 µm. The RIF-ETH 1:6 formulation did not produce particles with a suitable size for inhalation; the x_50_ and x_90_ particles far exceeded the limit value of less than 5 µm for inhalation.

For the RIF-MOX formulations, BM successfully produced a co-amorphous state but failed to reduce the particle size sufficiently for inhalation therapy (Table 11). Although the x_50_ is within the required range, the x_90_ value and the SPAN show unacceptable values. Here, only the particle size diameter (PSD) data of the BM 1:1 formulation are shown. Additional jet milling was applied to the mixtures in therapeutically relevant ratios (1:1.25 and 1:23.8) to generate particles with the required distributions for pulmonary formulations. The results show that via a combination of BM and jet milling, particles suitable for inhalation therapy could be obtained; the PSD is narrower, and the x_50_ and x_90_ values are reduced.

RIF-MOX PM showed particle sizes suitable for inhalation but with a slightly larger mean particle size (x_50_) and larger SPAN compared to the SD RIF-MOX formulations (Table 11).

Overall, BM proved unsuitable for generating inhalable particles, as it failed to produce fine particles and resulted in higher SPAN and SMD values compared to SD and jet milling. In contrast, SD yielded smaller particle size distributions (SPAN values below 1.93) and lower SMD values (below 1.96).

#### 3.2.3. Particle Morphology

**Figure 5:**
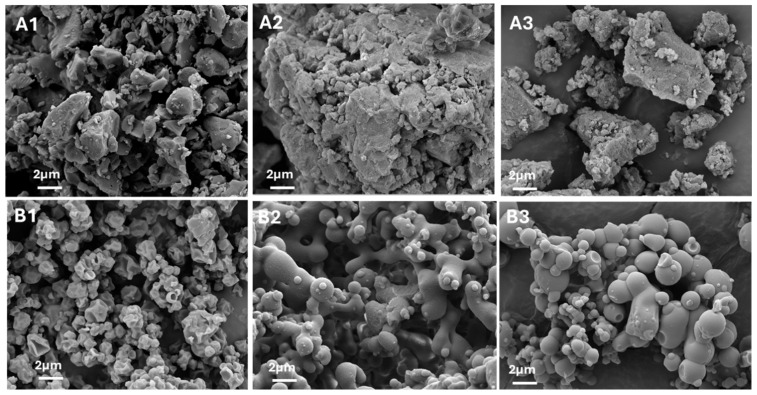
SEM images of RIF-ETH formulations in different molar ratios and prepared via milling (BM) or spray drying (SD): (**A1**) RIF-ETH 1:1 BM, (**B1**) RIF-ETH 1:1 SD, (**A2**) RIF-ETH 1:6 BM, (**B2**) RIF-ETH 1:6 SD, (**A3**) RIF-ETH 1:45 BM, and (**B3**) RIF-ETH 1:45 SD (image width 22.87 µm).

SEM images support our observations from the PSD measurements. All RIF-ETH BM samples (Figure 5(A1–A3)) show large, irregular, and partially agglomerated particles exceeding a size of 5 µm. SD of RIF-ETH 1:1 resulted in small corrugated spheres (Figure 5(B1)) with an adequate size for inhalation therapy. By contrast, the RIF-ETH 1:6 formulation did not produce particles with a suitable size for inhalation. SEM images in Figure 5(B2) provide the explanation; the particles appear to be fused together, and no single particles were produced, suggesting incomplete drying during the SD process. Only the RIF-ETH 1:45 formulation yielded particles suitable for inhalation, showing mostly separate, spherical particles with smooth surfaces (Figure 5(B3)).

**Figure 6:**
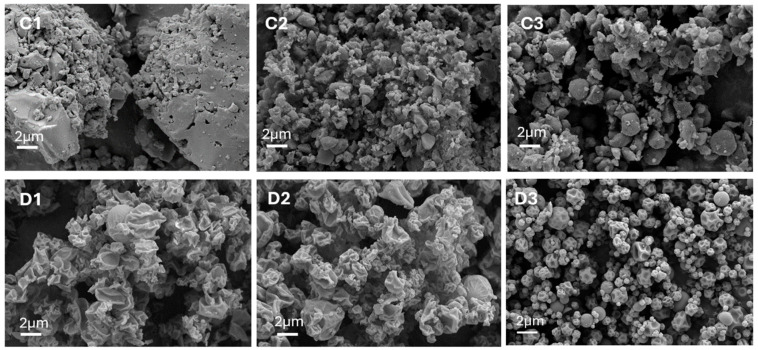
SEM images of RIF-MOX formulations in different molar ratios and prepared via ball milling (BM), co-milling (CM), or spray drying (SD): (**C1**) RIF-MOX 1:1 BM, (**D1**) RIF-MOX 1:1 SD, (**C2**) RIF-MOX 1:1.25 CM, (**D2**) RIF-MOX 1:1.25 SD, (**C3**) RIF-MOX 1:23.8 CM, and (**D3**) RIF-MOX 1:23.8 SD (image width 22.87 µm).

RIF-MOX 1:1 samples were only BM, but RIF-MOX 1:1.25 and 1:23.8 were BM and jet-milled. The SEM images clearly show size reduction due to jet milling, confirming the PSD analysis. Further SEM images show smaller irregular particles with more or less soft edges. Compared to the RIF-ETH formulations, SD of RIF-MOX produced spherical but shriveled particles, particularly pronounced at ratios of 1:1 and 1:1.25 (Figure 6(D1,D2)). The RIF-MOX 1:23.8 SD particles appeared to be less shriveled, resembling spheres with some dimples (Figure 6(D3)). The SD particles were smaller, with reduced SPAN and SMD values, indicating improved inhalation properties compared to BM and jet milling. The SEM images support this, showing large, agglomerated particles for BM RIF-MOX 1:1 (Figure 6(C1)), while smaller, irregularly shaped particles were observed for RIF-MOX 1:1.25 and 1:23.8 after jet milling (Figure 6(C2,C3)).

RIF MOX 1:1.25 and 23.8 PMs more resemble the CM samples, as initial API particles were too large and had to be jet-milled before blending. SEM images of the starting material and PMs are shown in the Supplementary Materials (Figures S3 and S4, respectively).

#### 3.2.4. Particle Stability

The stability evaluation in this study was limited to solid-state characterization using XRD to monitor the physical stability of the co-amorphous systems. RIF-MOX formulations are stable for a minimum of 3 months at ambient temperatures and dry conditions (Supplementary Materials, Figure S5).

### 3.3. Particle Performance

#### 3.3.1. API Distribution/API Content

Only COAMS prepared in therapeutically relevant molar ratios were evaluated for their aerosolization properties.

Oral doses required for TB treatment—450–600 mg for RIF and 800–2000 mg for MOX—are relatively high compared to the typical doses used in inhalation therapies for asthma or chronic obstructive pulmonary disease (COPD). While inhalation therapy offers the advantage of lower systemic dosing to achieve comparable therapeutic effects, TB drug doses remain substantial. As a general rule, inhalation doses are approximately one-tenth of oral doses or, based on our GP modeling, can be estimated assuming a 1.7% lung exposure for RIF and 32.5% lung exposure for MOX (considering a 600 mg oral dose for RIF, 1200 mg for MOX, and a 40% FPF).

Therefore, in line with our previous work, carrier-free formulations of CM and SD RIF-MOX at 1:1.25 and 1:23.8 molar ratios were tested and compared with their corresponding crystalline PMs. Carrier-free DPIs are preferred when high drug loads are required, as in the case of TB treatment [30].

For inhalation products, the acceptable limit for content uniformity is ±15% of the label claim. Accordingly, the mean drug content and its deviation were assessed using 10 samples of 25 mg each, corresponding to the fill weight of one capsule [30]. As shown in Table 12, all formulations complied with the content uniformity requirements. Inhalation formulations are typically considered homogenous when the relative standard deviation (RSD) of drug content is below 5% [31]. With the exception of the RIF-MOX 1:1.25 PM, all formulations demonstrated RSD values below this threshold. Therefore, all but the RIF-MOX 1:1.25 PMs can be regarded as sufficiently homogenous.

**Figure 7:**
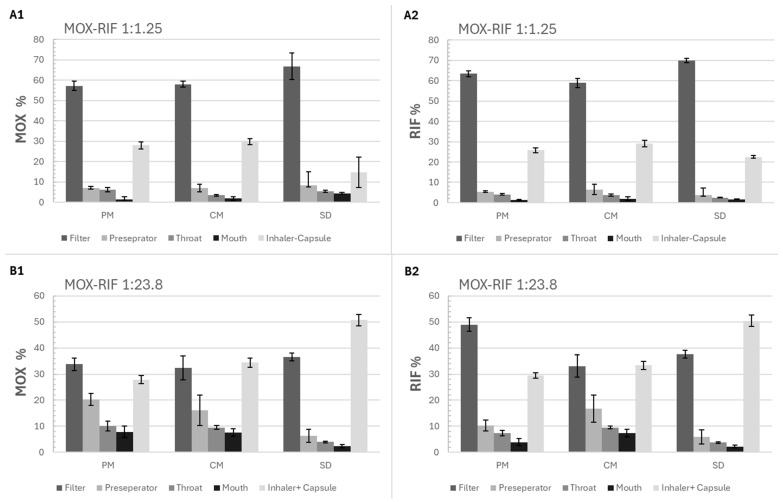
Percentage of RIF and MOX depositions in each stage of FSI (filter, preparator, throat, mouth, and inhaler + capsule) for the physical mixture (PM), spray-drying (SD), and co-milling (CM) formulations of 2 molar ratios (1:1.25 and 1:23.8), (**A1**) MOX percentage for the 1:1.25 formulations, (**A2**) RIF percentage for the 1:1.25 formulations, (**B1**) MOX percentage for the 1:23.8 formulations, (**B2**) RIF percentage for the 1:23.8 formulations, mean ± SD, *n* = 3.

Figure 7 shows the deposition of RIF and MOX in the different parts of the FSI. For the RIF-MOX 1:1.25 formulations, the same trends can be observed for both APIs. Comparing the different formulations, the SD formulation showed the highest deposition of MOX and RIF on the filter and the lowest in the inhaler and capsule. The PM and CM formulations show comparable deposition patterns independent of the preparation method.

#### 3.3.2. Aerodynamic Performance

The aerodynamic performance results (Figure 7 and Table 13) indicate that the EF for all RIF-MOX formulations is relatively low. For the RIF-MOX 1:23.8 formulations, EF values ranged from 49% to 70%, suggesting a substantial portion of the powder remained in the capsule and inhaler. In contrast, the RIF-MOX 1:1.25 formulations demonstrated improved performance, with EF values exceeding 70%. The highest EF was observed for the RIF-MOX 1:1.25 SD formulation.

Regarding the FPF, values of approximately 80% for MOX and 85–90% for RIF were achieved with the RIF-MOX 1:1.25 formulations, with the SD formulation again showing the best performance. This formulation also exhibited the highest FPM, delivering 5 mg MOX and 9.4 mg RIF (Table 13).

**Figure 8:**
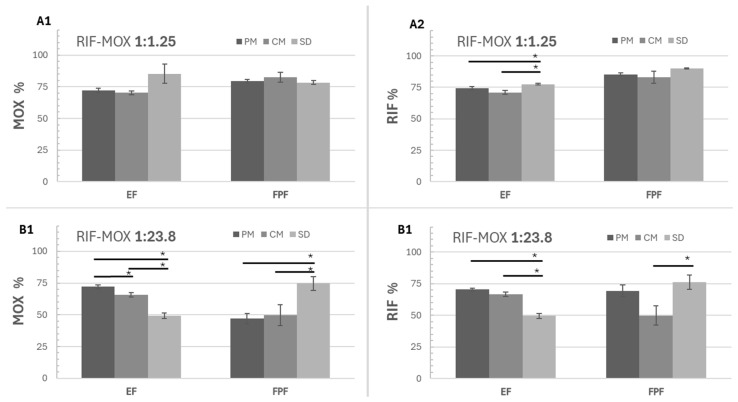
Emitted fraction (EF) and fine particle fraction (FPF) of MOX and RIF for the physical mixture (PM), spray-drying (SD), and co-milling (CM) formulations of 2 molar ratios (1:1.25 and 1:23.8), (**A1**) EF and FPF of MOX for the 1:1.25 formulations, (**A2**) EF and FPF of RIF for the 1:1.25 formulations, (**B1**) EF and FPF of MOX for the 1:23.8 formulations, (**B2**) EF and FPF of RIF for the 1:23.8 formulations, mean ± SD, *n* = 3, significant at * *p* ≤ 0.05.

For the RIF-MOX 1:23.8 formulations, the SD formulation displayed a significantly higher FPF (74.6% for MOX and 76.2% for RIF) compared to its CM and PM counterparts, although it had the lowest EF (49%). Nevertheless, the FPM of the SD formulation also remained the highest at 8.5 mg, compared to 6.6 mg for the CM and 7.1 mg for the PM formulations. Also notable is that overall variations within the tests were higher compared to the 1:1.25 formulations, indicated by overall higher standard deviations (Figure 7 and Figure 8).

In conclusion, among the tested RIF-MOX co-amorphous formulations, the SD variant demonstrated the most promising aerodynamic properties in terms of FPF and FPM. However, its EF remained relatively low, indicating potential for further optimization, such as the inclusion of excipients or process modifications to enhance powder emission. A comparison of therapeutic molar ratios revealed that the GP-modeled 1:23.8 ratio, although theoretically justified, resulted in extremely low RIF content—leading to an FPM below 0.8 mg, which is likely insufficient for therapeutic efficacy. This calls into question the practical relevance of the model-derived ratio. To achieve therapeutically relevant doses of RIF, another inhaler (e.g., RS01 DPI, Plastiape Spa, Osnago, Italy), together with an increased capsule size (size 0, which can hold up to 200 mg powder), could be used [40]. In contrast, the 1:1.25 ratio, based on the general assumption of inhalation doses being approximately one-tenth of oral doses, yielded more balanced drug delivery, with adequate lung deposition of both RIF and MOX and acceptable EDs. Interestingly, the SD formulation outperformed the CM and PM formulations in this study, which differs from previous findings with RIF-ETH combinations, where CM showed superior performance. Overall, the 1:1.25 molar ratio appears more suitable for inhalation-based TB therapy, combining feasible formulation design with therapeutic relevance.

#### 3.3.3. Dissolution Study

**Figure 9:**
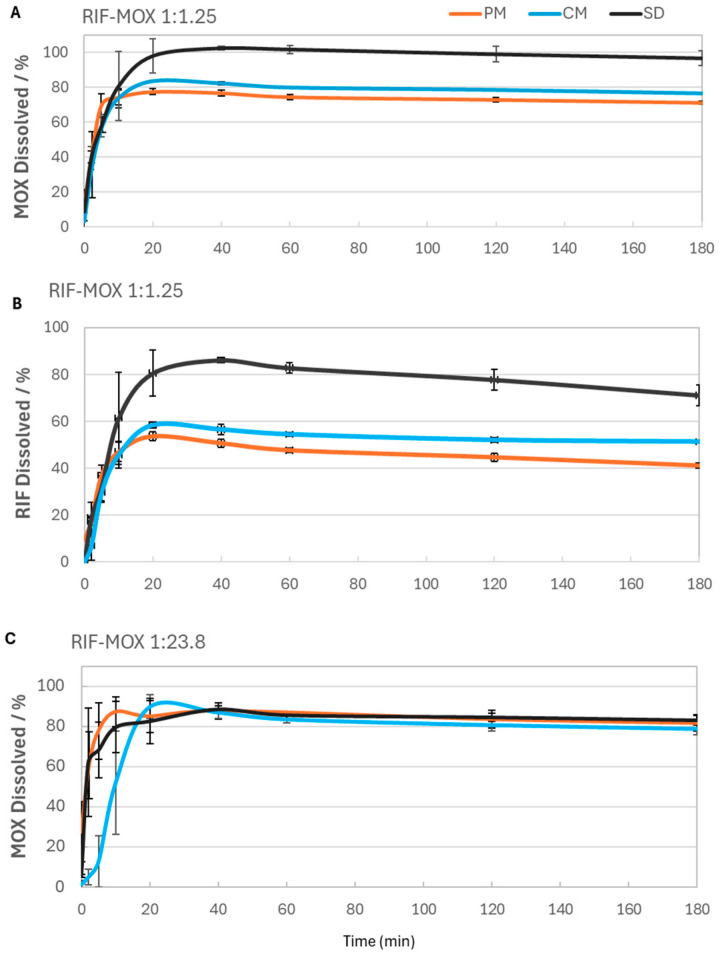
RIF-MOX formulation dissolution profiles over time for both RIF and MOX: (**A**) MOX dissolved for RIF-MOX 1:1.25, (**B**) RIF dissolved for RIF-MOX 1:1.25, and (**C**) MOX dissolved for RIF-MOX 1:23.8; mean ± SD, *n* = 3.

Unlike dissolution testing of oral formulations, there are no standardized protocols for pulmonary formulations. This includes the use of both dissolution fluid and an apparatus. In their review of dissolution testing, Radivojev et al. [41] compared the compositions of various solutions used in the literature. Wauthoz and Amighi [42] suggested that phospholipids, such as DPPC, can modulate the physicochemical properties of drug delivery and enhance drug permeability through the lung epithelium. Based on this hypothesis, DPPC has been included in the SLF by several research groups, e.g., [43]. The poor solubility of water-soluble APIs, such as budesonide, was demonstrated, whereas there were no changes in solubility noted for highly water-soluble APIs, such as salbutamol [44]. This increase in budesonide solubility was explained through reversible interaction with DPPC micelles, creating a thermodynamically stable isotropic solution. Looking first at the 1:1.25 formulations (Figure 9A,B), it is notable that RIF dissolves more slowly than MOX. The dissolution rate is directly proportional to drug solubility, meaning that poorly water-soluble compounds have slower dissolution rates compared to highly soluble drugs. Therefore, it is not surprising that MOX dissolves much faster than RIF. MOX reaches its maximum dissolution within 20 min and remains relatively stable over the 180 min period. Among the formulations, the SD sample—also exhibiting the highest FPF and FPM—achieved the most complete MOX release, reaching 100% dissolution. In contrast, the CM and PM formulations reached a maximum of approximately 80% dissolved MOX.

RIF showed a slower dissolution profile, with peak concentrations occurring between 20 and 30 min, preceded by a gradual increase. The increase was not much slower than for the highly water-soluble MOX, which may be due to the presence of DPPC in SLF. The SD formulation again performed best, achieving around 86% dissolution, while the CM and PM formulations dissolved to a lesser extent (58% and 53%, respectively). From 30 to 180 min, a slight decline in RIF concentration was observed, likely due to degradation in aqueous media. Chromatograms (provided in the Supplementary Materials, Figure S6) confirmed the presence of degradation peaks, primarily corresponding to RIF quinone—a degradation product that, while less active, retains some antibacterial activity and can revert to RIF at body temperature [45].

In the 1:23.8 formulations, MOX dissolved even more rapidly, with peak concentrations reached within 10 min (Figure 9C). In this ratio, the co-amorphous formulations (SD and CM) again outperformed the crystalline PMs in terms of dissolution rate, particularly in the early stages. This may be attributed to the significantly higher MOX content and the greater variability in the PMs’ dissolution behavior, especially within the first 10 min. RIF concentrations in the 1:23.8 formulations, however, were below the limit of quantification and could not be reliably assessed..

In the PBPK-modeled therapeutically relevant RIF-MOX combination (1:23.8 molar ratio), RIF is present in a significantly lower amount compared to MOX, primarily because the estimated lung exposure required for effective oral RIF treatment is very low—only about 1.7%. The fact that relatively high oral doses are needed to achieve this low yet therapeutically effective lung concentration underscores the potential benefit of direct pulmonary delivery. For drugs like RIF, inhalation could offer a more efficient strategy to enhance local efficacy while minimizing systemic side effects. Notably, high-dose RIF regimens (up to 35 mg/kg) are also being considered in clinical settings, which contrasts with the lower dose used in our study (10 mg/kg, or 600 mg total). To address the imbalance between RIF and MOX in the formulation and enhance its manufacturability and analytical quantifiability, future work should focus on increasing the RIF content in the COAMS, also considering high-dose RIF delivery. Ultimately, optimizing the RIF-MOX ratio could support the development of a more effective and scalable inhalable therapy for pulmonary infections.

## 4. Conclusions

This study demonstrated how in silico tools can be used to develop COAMS for pulmonary formulations of APIs used in oral formulations. PBPK modeling was used to determine the relative ratios, and ML predicted the COAMS from the APIs. Experimental validation of these predictions showed that SD and BM were both suitable for generating RIF-MOX co-amorphous formulations, as predicted by our in-house-built predictive ML model. SD produced better results in terms of desirable aerodynamic properties, particle morphology, and dissolution. API ratios close to 1:1 produced more stable COAMS formation and more efficient aerosol performance. However, the study also showed that COAMS may not be ideal for APIs where the therapeutically relevant molar ratio in the lung differs markedly from the predicted 1:1 ratio. For example, RIF-MOX at a ratio of 1:23.8 is not ideal because the excess MOX leads to increased variability and reduced RIF deposition in the lung. In these cases, alternative delivery methods for the pulmonary administration of drug combinations must be considered.

## Figures and Tables

**Table 1:** Tissue-to-plasma partition coefficients (Kp values) of the different compartments used in the simulation.

Compartment	Kp Value—RIF	Kp Value—ETH	Kp Value—MOX
Lung	0.14	7.92	4.9
Adipose	0.28	1.22	0.52
Muscle	0.15	9.14	2
Liver	0.29	8.65	5.69
Spleen	0.19	8.75	4.01
Heart	0.14	7.48	2.88
Brain	0.16	8.9	0.64
Kidney	0.24	8.07	6.26
Skin	0.19	6.26	1.74
ReproOrg	0.24	8.07	6.33
RedMarrow	0.3	3.15	0.75
YellowMarrow	0.3	3.15	0.75
RestOfBody	5	46	17.55

**Table 2:** Input parameter GP. bp ratio: blood-to-plasma ratio, fu: fraction unbound, Vd_ss_: volume of distribution at steady state, P_eff_: effective permeability, CL_iv_: intravenous clearance (systemic clearance), CL_R_: renal clearance [22].

Parameter (Unit)	Value—RIF	Value—ETH	Value—MOX	Reference
Dose (mg)	300	1974.6	200	[22,23,24]
MW (g/mol)	822.94	204.31	401.44	GP
bp ratio	0.67	0.94	1.14	Refs. [22,25], GP
fu	0.15	0.75	0.52	Ref. [22], GP, ref. [24]
Vd_ss_ (L/kg)	0.42	6.1	1.9	Refs. [22,23,26]
P_eff_ (×10^−4^ cm/s)	1.3	0.67	1.45	Optimized GP
CL_iv_ (L/h)	8.307	41.9	13.15	Refs. [22,26,27]
CL_R_ (L/h)	1.5	32.4	2.54	Refs. [22,26,27]

**Table 3:** Mean anthropometric parameters of the study population.

Parameter (Unit)	Value—RIF	Value—ETH	Value—MOX
Age (years)	30	39.1	33.6
Body weight (kg)	85.5	79.3	81.5
Body height (cm)	176.4	172.6 (estimated)	182.2
Percentage male	100	57	100
Race/Sex	1 Caucasian	6 white female, 3 black males, 5 while males	45 Caucasian

**Table 4:** The different API-API combinations, molar ratios, and formulations for analysis.

API-API Combination	Formulation	Molar Ratio	Comment
RIF-ETH	BM, CM, SD *	1:1	ML-predicted
RIF-ETH	BM, SD	1:6	The literature
RIF-ETH	BM, SD	1:45	PBPK modeling
RIF-MOX	BM, SD	1:1	ML-predicted
RIF-MOX	PM, CM, SD	1:1.25	The literature
RIF-MOX	PM, CM, SD	1:23.81	PBPK modeling

* Data presented in previous paper [20].

**Table 5:** Chromatographic conditions for HPLC analysis of RIF and MOX.

Parameter	Condition
Column	Waters Acquity UPLC HSS Cyano (2.1 × 100 mm, 1.8 µm particle size), 25 °C
Mobile phase	A: 10 mM ammonium formate buffer (0.630 g/L), pH 4.0 (adjusted with diluted formic acid solution) B: 0.1% formic acid in methanol
Gradient program	0.0–1.5 min: 95% A 2.0 min: 50% A 6.0–7.0 min: 10% A 8.0 min: Return to 95% A Total run time ≈ 12 min
Flow rate	0.35 mL/min
Injection volume	1 µL
Sample manager temp.	10 °C
Detection wavelengths	295 nm (MOX); 335 nm (RIF)

**Table 6:** PK-parameters, maximum plasma concentrations (C_max_) and area under the curve (AUC), of the clinical trials (Obs.) compared to the ones from the simulation (Sim.) with the percentage prediction error (%PE).

	RIF			ETH			MOX		
	Obs.	Sim.	%PE	Obs.	Sim.	%PE	Obs.	Sim.	%PE
C_max_ (µg/mL)	5.81	6.01	3.44	3.54	3.56	0.68	1.16	1.20	3.48
AUC_0-t_ (µg-h/mL)	31.02	33.67	8.54	27.80	28.52	2.57	11.88	11.25	5.27
AUC_0-inf_ (µg-h/mL)	31.02	34.23	10.35	30.76	30.06	2.27	14.57	13.58	6.75

**Table 7:** PBPK-model-estimated lung exposure for RIF, ETH, and MOX.

API	Estimated Lung Exposure (%)
RIF	1.7
ETH	13
MOX	32.5

**Table 8:** Inhalation doses of RIF, ETH, and MOX based on predicted estimated lung exposure and 40% FPF.

API	Inhalation Dose [mg] (Model)	Inhalation Dose [mg] (Literature)
RIF	25.65	150
ETH	390	300
MOX	325	100

**Table 9:** Molar ratio of RIF-ETH and RIF-MOX inhalation doses.

API-API	Molar Ratio (Model)	Molar Ratio (Literature)
RIF-ETH	1:45	1:6
RIF-MOX	1:23.8	1:1.25

**Table 10:** Particle size distribution of RIF–ETH formulations.

Formulation	X_10_ µm	X_50_ µm	X_90_ µm	SPAN	SMD µm
RIF-ETH 1-1 SD	1.59 ± 0.50	2.1 ± 0.7	5.3 ± 15.34	0.98 ± 0.02	2.17 ± 0.09
RIF-ETH 1-1 BM	0.99 ± 0.05	3.85 ± 0.51	10.16 ± 1.71	64.23 ± 6.87	2.38 ± 0.36
RIF-ETH 1:6 SD	1.40 ± 0.05	6.68 ± 0.22	22.77 ± 1.82	3.20 ± 0.19	3.28 ± 0.11
RIF-ETH 1:6 BM	2.31 ± 0.40	23.06 ± 5.94	114.85 ± 32.8	4.93 ± 0.75	6.13 ± 0.94
RIF-ETH 1:45 SD	1.30 ± 0.04	3.77 ± 0.23	7.23 ± 0.28	1.57 ± 0.04	2.63 ± 0.09
RIF-ETH 1:45 BM	1.69 ± 0.12	9.84 ± 2.12	53.13 ± 1.10	5.50 ± 1.27	4.44 ± 0.42

**Table 11:** Particle size distribution of RIF–MOX formulations.

Formulation	X_10_ µm	X_50_ µm	X_90_ µm	SPAN	SMD µm
RIF-MOX 1:1 SD	1.01 ± 0.02	2.82 ± 0.07	4.94 ± 0.14	1.39 ± 0.01	1.88 ± 0.04
RIF-MOX 1:1 BM	0.98 ± 0.02	4.31 ± 0.18	20.00 ± 2.44	4.40 ± 0.39	2.35 ± 0.06
RIF-MOX 1:1.25 PM	0.94 ± 0.02	3.96 ± 0.16	7.30 ± 0.26	1.61 ± 0.03	2.14 ± 0.06
RIF-MOX 1:1.25 SD	1.05 ± 0.05	2.99 ± 0.18	5.38 ± 0.32	1.45 ± 0.02	1.96 ± 0.08
RIF-MOX 11.25 CM	1.05 ± 0.05	3.84 ± 0.25	7.27 ± 0.28	1.62 ± 0.06	2.24 ± 0.10
RIF-MOX 1:23.8 PM	1.32 ± 0.01	5.94 ± 0.15	11.14 ± 0.24	1.65 ± 0.02	2.90 ± 0.04
RIF-MOX 1:23.8 SD	0.78 ± 0.05	3.18 ± 0.33	6.91 ± 0.70	1.93 ± 0.04	1.82 ± 0.13
RIF-MOX 1:23.8 CM	1.00 ± 0.08	4.92 ± 0.87	12.05 ± 0.83	2.29 ± 0.27	2.43 ± 0.23

**Table 12:** Mean drug content (%) and mixing homogeneity (deviation in mean drug content, relative standard deviation, (%)) for RIF and MOX within the 3 formulations RIF-MOX PM, RIF-MOX SD, and RIF-MOX CM (*n* = 10).

	Mean Drug Content RIF/%	Mixing Homogeneity RIF/%	Mean Drug Content MOX/%	Mixing Homogeneity MOX/%
RIF + MOX PM 1:1.25	92.8 ± 10.9	11.8	86.8 ± 9.7	11.2
RIF + MOX SD 1:1.25	95.7 ± 2.5	2.6	89.0 ± 3.0	3.3
RIF + MOX CM 1:1.25	90.3 ± 2.5	2.7	89.2 ± 3.0	3.4
RIF + MOX PM 1:23.8	91.5 ± 3.2	3.5	93.6 ± 2.4	2.5
RIF + MOX SD 1:23.8	97.1 ± 4.8	4.9	103.2 ± 5.3	5.0
RIF + MOX CM 1:23.8	87.4 ± 3.4	3.9	95.1 ± 3.0	3.1

**Table 13:** EF, FPM, and FPF RIF-MOX for both RIF and MOX.

Formulation	MOX			RIF		
	EF %	FPM mg	FPF%	EF %	FPM mg	FPF %
RIF-MOX 1:1.25 PM	71.9 ± 1.7	5.1 ± 0.4	79.5 ± 1.4	74.2 ± 1.2	9.2 ± 0.5	85.4 ± 1.1
RIF-MOX 1:1.25 CM	70.3 ± 1.5	4.9 ± 0.3	82.6 ± 3.9	71.0 ± 1.5	7.7 ± 0.6	83.0 ± 4.8
RIF-MOX 1:1.25 SD	85.2 ± 7.6	5.0 ± 0.2	78.3 ± 1.5	77.5 ± 0.7	9.4 ± 0.4	90.0 ± 0.6
RIF-MOX 1:23.8 PM	72.1 ± 1.6	7.1 ± 0.7	47.0 ± 4.2	70.6 ± 1.1	0.8 ± 0.05	69.4 ± 4.5
RIF-MOX 1:23.8 CM	65.6 ± 1.8	6.6 ± 1.0	49.5 ± 8.3	66.6 ± 1.6	0.5 ± 0.07	49.8 ± 7.6
RIF-MOX 1:23.8 SD	49.2 ± 2.2	8.5 ± 0.3	74.6 ± 5.3	49.5 ± 2.1	0.6 ± 0.01	76.2 ± 5.5

## Data Availability

The original contributions presented in this study are included in the article/Supplementary Materials. Further inquiries can be directed to the corresponding author.

## Supplementary Materials

The following supporting information can be downloaded at https://www.mdpi.com/article/10.3390/pharmaceutics17101339/s1, Figure S1: XRD pattern of RIF and ETH starting material and RIF-ETH 1:1.6 co-milled and spray dried sample (A) and RIF-ETH 1:45 co-milled and spray dried sample (B).). Figure S2: XRD patterns of RIF and MOX starting material and the physical mixtures (PMs) or RIF-MOX 1:1.25 and RIF-MOX 1:23.8. Figure S3: SEM images of rifampicin starting material (A1), ethambutol starting material (B1) and moxifloxacin starting material (C1). Figure S4: SEM images of physical mixtures (PM) or RIF-MOX in therapeutic relevant molar ratios (A1) 1:1.25 and (B1) 1:23.8. Figure S5: XRD pattern after 3 month stability study for RIF-MOX COAMS (A) co-milled 1:1 (RIF-MOX BM 1:1), (B) spray dried 1:1 (RIF-MOX SD 1:1), (C) co-milled 1:1.25 (RIF-MOX CM 1:1.25), (D) spray dried 1:1.25 (RIF-MOX SD 1:1.25), (E) co-milled 1:23.8 (RIF-MOX CM 1:23.8), (F) spray dried 1:23.8 (RIF-MOX SD 1:23.8). Figure S6: Exemplary, spectrum index plot of HPLC chromatograms for RIF-MOX CM 1:1.25 dissolution, 60 min.
