# Supplementary material for: Development of Co-Amorphous Systems for Inhalation Therapy—Part 2: In Silico Guided Co-Amorphous Rifampicin–Moxifloxacin and –Ethambutol Formulations
Source: Pharmaceutics. 2025 Oct 16;17(10):1339. doi: 10.3390/pharmaceutics17101339 (PMC12567144; doi:10.3390/pharmaceutics17101339)
Supplement: Supplementary file 1 [file pharmaceutics-17-01339-s001.zip › pharmaceutics-3889441-supplementary.pdf]

pharmaceutics MDPI

Supplementary Information

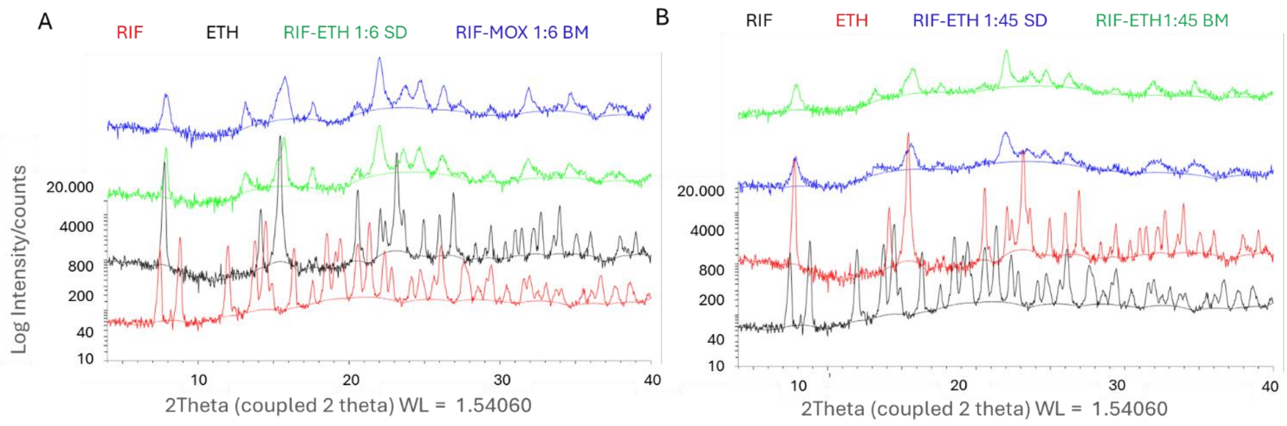

Figure S1: XRD pattern of RIF and ETH starting material and RIF-ETH 1:1.6 co-milled and spray dried sample (**A**) and RIF-ETH 1:45 co-milled and spray dried sample (**B**).

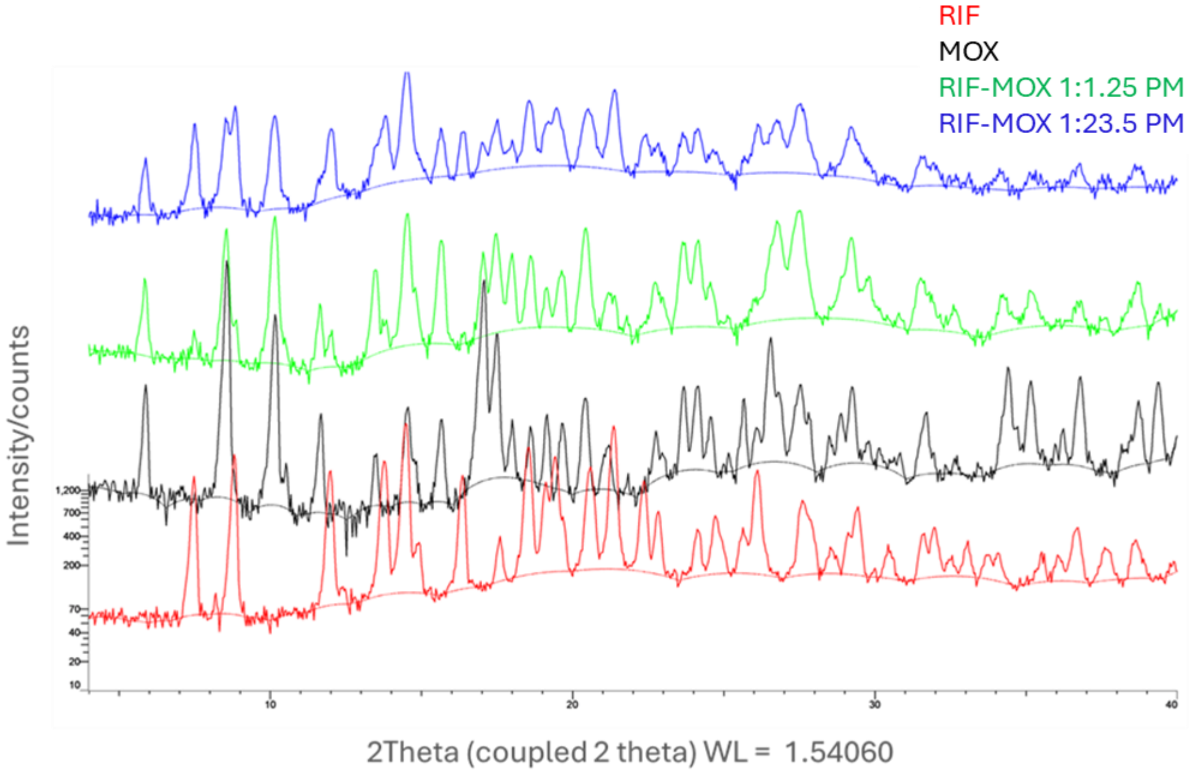

Figure S2: XRD patterns of RIF and MOX starting material and the physical mixtures (PMs) or RIF-MOX 1:1.25 and RIF-MOX 1:23.8.

pharmaceutics

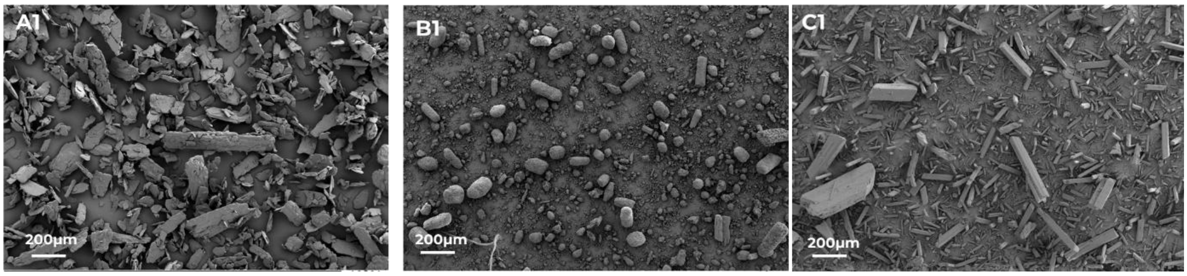

Figure S3: SEM images of rifampicin starting material (**A1**), ethambutol starting material (**B1**) and moxifloxacin starting material (**C1**).

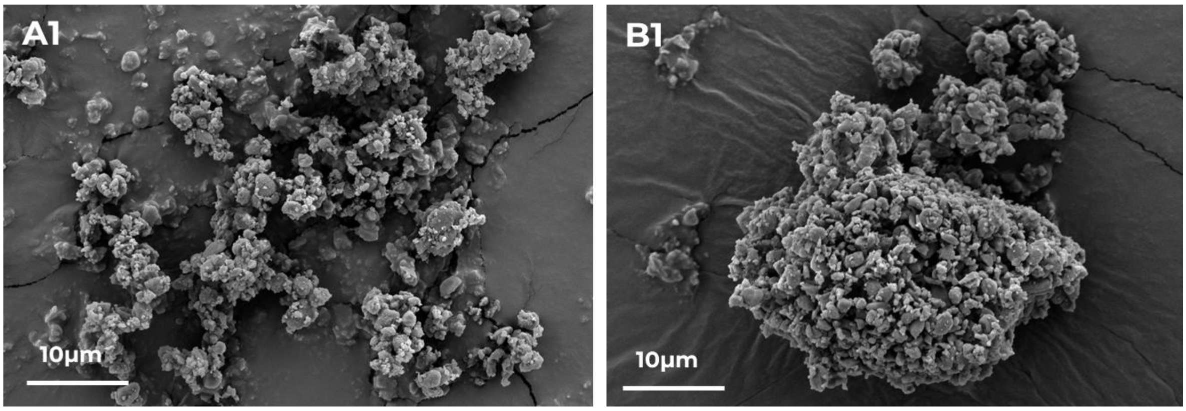

Figure S4: SEM images of physical mixtures (PM) or RIF-MOX in therapeutic relevant molar ratios (A1) 1:1.25 and (B1) 1:23.8.

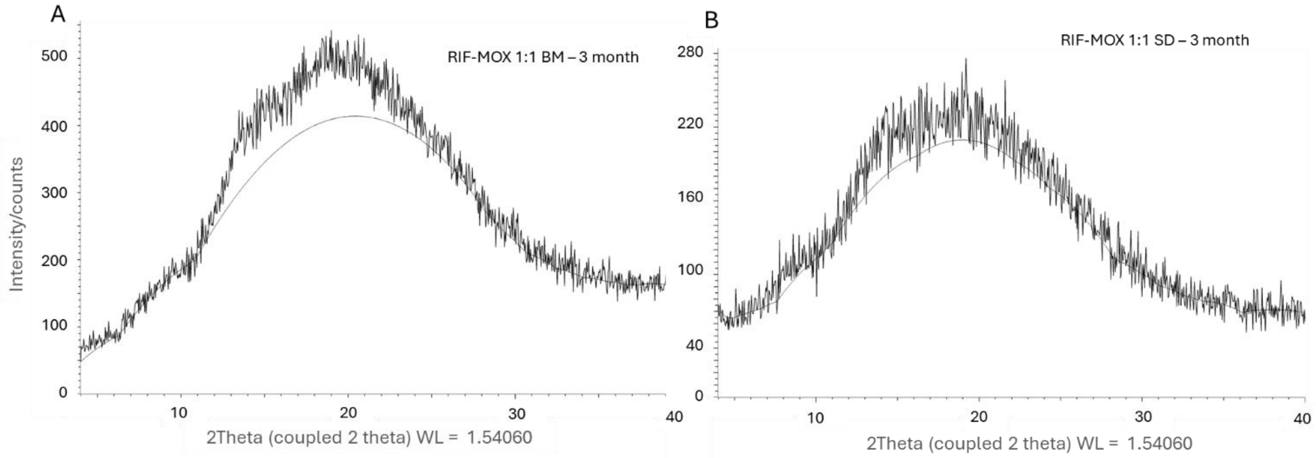

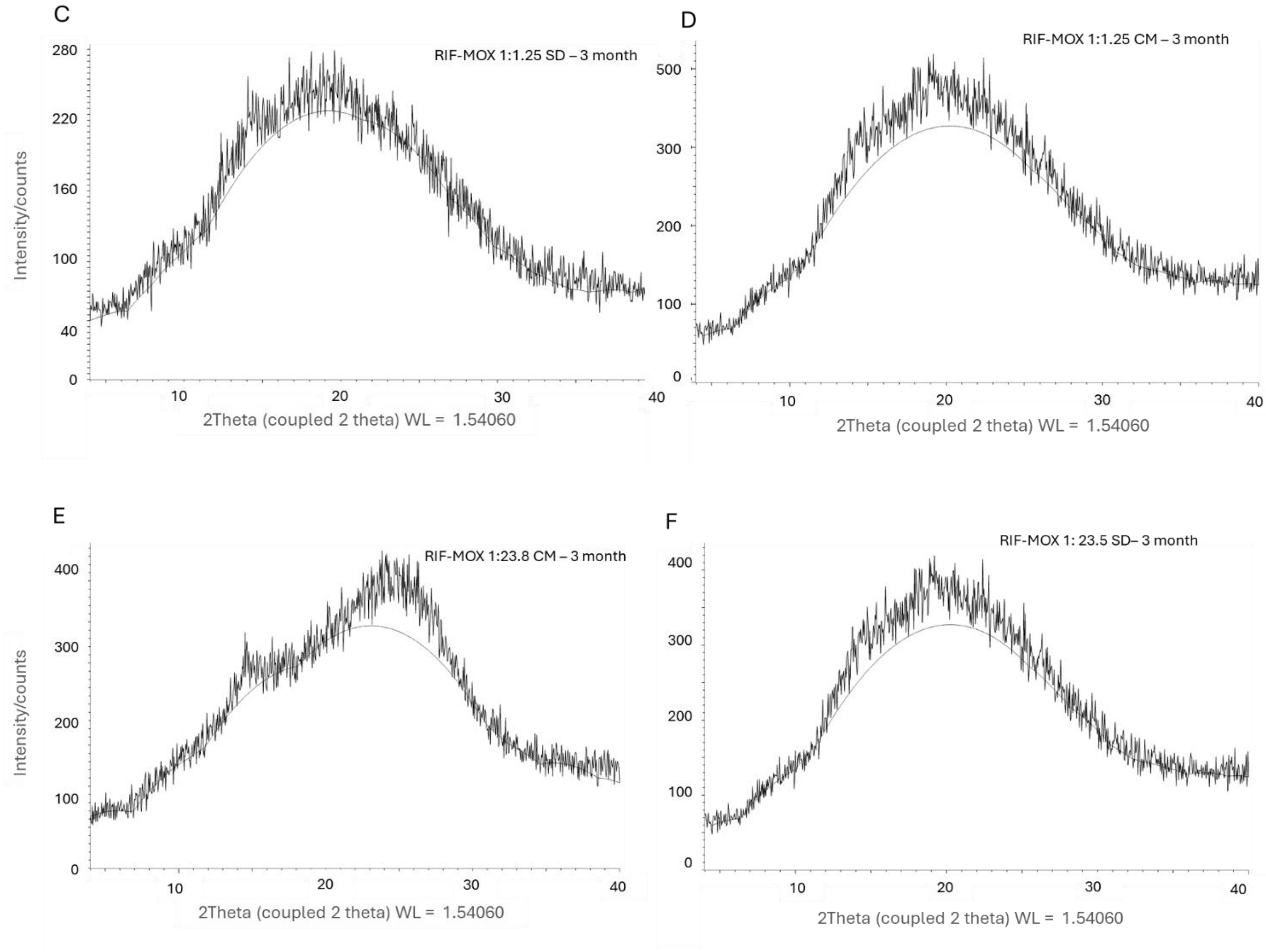

Figure S5: XRD pattern after 3 month stability study for RIF-MOX COAMS (**A**) co-milled 1:1 (RIF-MOX BM 1:1), (**B**) spray dried 1:1 (RIF-MOX SD 1:1), (**C**) co-milled 1:1.25 (RIF-MOX CM 1:1.25), (**D**) spray dried 1:1.25 (RIF-MOX SD 1:1.25), (**E**) co-milled 1:23.8 (RIF-MOX CM 1:23.8), (**F**) spray dried 1:23.8 (RIF-MOX SD 1:23.8).

pharmaceutics

MDPI

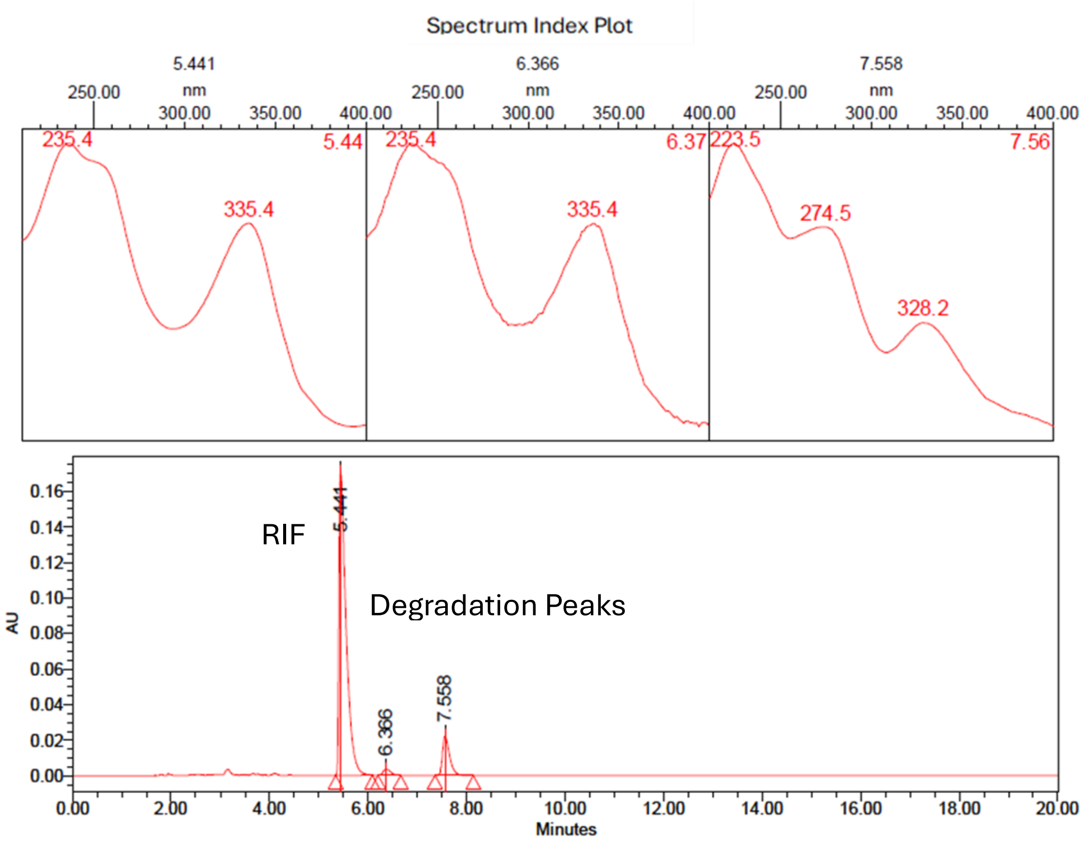

Figure S6: Exemplary, spectrum Index plot of HPLC Chromatograms for RIF-MOX CM 1:1.25 dissolution 60 minutes, Δ mark start and end of the peaks.
